# Chemoradiation with capecitabine and mitomycin-C for stage I-III anal squamous cell carcinoma

**DOI:** 10.1186/1748-717X-9-124

**Published:** 2014-05-29

**Authors:** Govin Thind, Bal Johal, Matthew Follwell, Hagen Fritz Kennecke

**Affiliations:** 1 Royal College of Surgeons in Ireland, BC Cancer Agency, Fraser Valley Cancer Center, Surrey, BC, Canada; 2 Division of Medical Oncology, BC Cancer Agency, Fraser Valley Cancer Center, Surrey, BC, Canada; 3 Division of Radiation Oncology, Simcoe Muskoka Regional Cancer Centre, Barrie, ON, Canada; 4 Division of Medical Oncology, British Columbia Cancer Agency – Vancouver Center, Vancouver, BC V5Z 4E6, Canada

**Keywords:** Overall survival, Toxicity, Squamous cell carcinoma, Patient outcomes, Chemoradiation

## Abstract

**Background:**

Standard therapy for patients with stage I-III squamous cell carcinoma (SCC) of the anal canal is chemo-radiotherapy with 5-fluorouracil (5-FU) and mitomycin C (MMC). While there is limited published evidence to substitute capecitabine (CAP) for 5-FU, the objectives of the study were to describe the toxicity, dose intensity and outcomes of a sequential cohort of patients treated with chemo-radiotherapy with CAP and MCC in a population-based setting.

**Methods:**

Patients with stage I-III malignancies of the anal canal referred between February 2010 and March 2012 were included. Dose intensity was calculated by comparing delivered versus planned radiation and chemotherapy treatments and toxicity was retrospectively graded according to standard protocol-specified criteria.

**Results:**

Among 66 eligible patients, median planned dose of radiation was 51.9 Gy over 5.5 weeks, range 25.0 to 63 Gy, and dose intensity was 98%. Median delivered dose of MCC delivered was 12 mg/m^2^ on day one, week one while median CAP dose was 825 mg/m^2^ twice daily on radiation days. CAP dose reductions due to toxicity were recorded for 13 patients (20%). Median follow-up was 20 months and 94% of patients with squamous cell histology had no evidence of relapse.

**Conclusions:**

Chemo-radiation with CAP plus MMC is well tolerated and may be a reasonable consideration for patients with stage I-III SCC of the anal canal. A range of planned radiation dose was observed and longer follow-up is necessary to ensure that patients who received lower doses of radiation have similar outcomes to those who received larger doses.

## Background

Carcinoma of the anal canal is a relatively uncommon malignancy, accounting for approximately 2.5 percent of all gastrointestinal cancers [1]. The incidence of this disease has been on the rise for the past few decades [2], which is thought to be due, in part, to the increased sexual transmission of human papilloma virus (HPV) [3,4].

Squamous cell carcinoma (SCC) of the anal canal remains the only carcinoma of the gastrointestinal tract that is curable without the need for definitive surgery, with 5 year survival rates nearing 90% for early stage disease [2,3,5,6]. Treatment regimens for SCC of the anal canal have evolved over the past decades, and studies have included comparisons of radiotherapy alone versus chemoradiation [7-9]; determining treatment benefits of mitomycin C (MMC) [10,11]; and comparisons of MMC with cisplatin [6,12-15]. The accepted current standard regimen for patients with stage I-III SCC of the anal canal is radiotherapy (50.4 Gy) with concurrent infusional 5-fluorouracil (5-FU) administered days one through four during weeks one and five and MMC administered on day one of week one. Substitution of MMC for cisplatin on days one and 29 of radiation has been shown equally efficacious and associated with significantly less hematologic toxicity [6].

Capecitabine (Cap) is an oral fluoropyrimidine shown to be equivalent to infusional 5-FU when given concurrently with pelvic irradiation in the neo-adjuvant treatment of rectal adenocarcinoma [16-18]. Unlike 5-FU, which is intravenously infused, Cap is orally administered which provides resource benefits as it is more convenient for patients and staff and does not require the use of a central venous infusional device. There is only limited evidence to support the substitution of infusional 5-FU for capecitabine in the treatment of SCC of the anal canal. In a previously published phase II study, thirty-one patients with stage I-III SCC of the anal canal were treated with continuous radiation, Cap on radiation days and MMC on day 1 [19]. A total of 24 (77%) of patients had a complete clinical response after four weeks, while 3 (16%) had a partial response. Three locoregional relapses occurred during the follow up period (median of 14 months) [19].

In this study, a retrospective chart review was conducted of a sequential cohort of patients with stage I-III SCC of the anal canal treated with standard radiation and concurrent Cap and MMC according to a standard province-wide protocol [20]. The objectives of the study were to: (1) describe the dose intensity of radiation, Cap and MMC by comparing the planned versus the delivered dose of radiation, Cap and MMC; (2) describe treatment-related patient toxicities and (3) describe the early outcomes of therapy.

## Methods

### Treatment of SCC of the anal canal

Patients were treated at one of five cancer treatment centers throughout the province of British Columbia (BC), a Canadian province with a population of 4.4 million. The BC Cancer Agency (BCCA) is responsible for funding all systemic cancer therapy and is the sole provider of radiotherapy in BC. All patients in the province who require radiation therapy for a diagnosis of SCC anal cancer are referred to the BCCA for consultation and treatment delivery.

Chemoradiation was delivered according to GICART, a standardized protocol, introduced in February 2010 and posted on the BCCA website [20]. Eligibility criteria for this therapy include a diagnosis of stage I-III squamous cell or cloacogenic carcinoma of the anal canal and ECOG performance status of less than or equal to 2. Patients also need to have an adequate marrow reserve (ANC greater than or equal to 1.5 × 10^9^/L, platelets greater than 100 × 10^9^/L), with adequate renal (creatinine less than or equal to 1.5 × ULN) and liver function (bilirubin less than or equal to 26 μmol/L; AST/Alkaline Phosphatase less than or equal to 5 × ULN).

Patients treated under the GICART protocol received a combination of chemotherapy and radiotherapy. Continuous radiation at a dose of 50.4 Gy in 28 fractions over 5 1/2 weeks was recommended. Chemotherapy with Cap was delivered twice a day at a dose of 825 mg/m^2^ on days that radiotherapy was administered (days 1–5, 8–12, 15–19, 22–26, 29–33, 36–40), to a total daily dose of 1650 mg/m^2^. As specified in the GICART protocol, Cap was administered orally with food, with the second dose administered 10–12 hours after the first. MMC is administered on day one week one intravenously, at a dose of 12 mg/m^2^ to a maximum dose of 20 mg.

### Study cohort selection and data extraction

All SCC anal cancer patients diagnosed at BCCA and treated with GICART protocol from the time of its introduction in February 1, 2010 until March 29, 2012 were included in this study.

The charts from all SCC anal canal patients in this cohort were used to determine variations in chemotherapy doses, differences in radiation dosing and type of radiation technique delivered to each patient. Patient outcomes including relapses and deaths were documented until April 2013. To determine the levels of toxicity experienced by each patient in the cohort, a review was conducted of all the narratives dictated by the oncologist assigned to each patient. Nausea, vomiting, stomatitis and diarrhea toxicities were graded according to the GICART protocol. Dermatitis data was extrapolated from the narratives provided by the physicians and a graded according to the Dermatitis Grading Scale from the BCCA Care of Radiation Skin Reactions standard [21]. The most severe toxicity described for each patient was used in each case. For example, if a patient had grade 3 diarrhea but was experiencing grade 4 levels of nausea, they were classified as experiencing a grade 4 toxicity.

### Sources of research support

The Provincial Pharmacy Database was used to identify eligible patients, and patient and tumor characteristics were identified through the BCCA Gastrointestinal Cancer Outcomes Unit (GICOU). The GICOU database prospectively documents standard pathologic and clinical criteria of patients referred to the BCCA. The study was conducted as a quality assurance initiative and was reviewed by the BCCA-University of British Columbia Research Ethics Board.

## Results

### Patient characteristics

The characteristics and diagnostic status of the 66 patients included in this study are shown in Table 1. The majority of patients were female (62%), HIV negative (99%), non-smokers (74%) and non-drinkers (74%). Most patients (n = 61, 93%) presented with an ECOG status between 0–1, while three (5%) presented with an ECOG status of 2 and two patients (3%) had an ECOG status of 3. Staging was varied, as 26 patients (39%) presented with a stage I tumor, 15 (23%) presented with a stage II tumor and 25 (38%) presented with a stage III tumor.

**Table 1:** Characteristics of patients included in study (n = 66)

**Characteristics**	**Number of patients (percent)**
**Median age**	60 years (44–82)
**Sex**	
** Male**	25 (38%)
** Female**	41 (62%)
**HIV status**	
** Positive**	1 (2%)
** Negative**	65 (99%)
**Smoking status**	
** Smoker (have ever)**	17 (26%)
** Non-smoker**	49 (74%)
**Alcohol consumption**	
** Drinker**	17 (26%)
** Non-drinker**	49 (74%)
**ECOG status**	
** 0**	42 (64%)
** 1**	19 (29%)
** 2**	3 (5%)
** 3**	2 (3%)
** 4**	0 (0%)
**Stage of tumor**	
** I**	26 (39%)
** II**	15 (23%)
** III**	25 (38%)
** IV**	0(0%)
**Histology**	
** Squamous cell carcinoma**	49 (74%)
** Squamous carcinoma (keratinizing)**	8 (12%)
** Squamous cell carcinoma (basaloid)**	5 (8%)
** Tubulovillous adenoma**	2 (3%)
** Adenocarcinoma**	2 (3%)

Characteristics of all 66 patients included in the study are shown above, both as a total number and as a percentage of study participants.

Histologically, the tumours were classified as follows: of 66 patients, 62 (94%) had squamous cell carcinoma of which eight were described as keratinizing, and five as basaloid. Two patients (3%) had tubulovillous adenoma, and an additional two patients (3%) had adenocarcinoma. All 66 patients were included in the dose intensity and toxicity analysis, while only patients with SCC (62) were included in the outcome analysis.

### Treatment dose intensity

All patients included in the study initiated therapy with both capecitabine and mitomycin C. The doses administered are shown in Table 2, along with the number of dose reductions, increases, omissions, and discontinuations for each therapy administered.

**Table 2:** Chemotherapy dosings (n = 66) as a total number and as a percentage

**Capecitabine dose administered**	**Number of patients (percent)**
**Number of patients starting at full dose (750–825 mg/m**^**2**^**)**	
**- No subsequent dose reduction**	59 (78%)
**- Dose reduction**	13 (22%)
**- Discontinuation**	0 (0%)
**Number of patients starting at dose level −1 (500–749 mg/m**^**2**^**)**	
**- Received**	7 (11%)
**- Dose reduction**	0 (0%)
**- Discontinuation**	0 (0%)
**Mean weeks of capectiabine**	5.5 (2.5-6)
**Mitomycin dose administered**	**Number of patients (percent)**
**- Full dose (12 mg/m**^**2**^**)**	63 (95%)
**- Dose level −1**	3 (5%)

The capecitabine and mitomycin dose administered to patients in the study, both as a total number and as a percentage of study participants.

Patients received a median dose of 825 mg/m^2^ of Cap administered twice daily on radiation days. Dose reductions were recorded for 13 patients (20%) due to apparent toxicity during treatment. There were no recorded increases, omissions or discontinuations for this drug. The median duration of therapy of capecitabine was five and a half weeks on week-days, with a treatment range from three to six weeks. Seven patients initiated therapy at a lower dose of Cap (500–749 mg/m^2^) due to varying issues ranging from patient comorbidities, previous chemotherapy treatment, and the use of therapy purely for palliative purposes. None of the patients starting at this lower dose required a subsequent dose reduction.

The median dose of MCC delivered was 12 mg/m^2^ on day one, week one. Pre-planned dose reductions were recorded for three (5%) of patients, of which two cases were due to patient co-morbidities and one was due to an infusion reaction.

The radiation dose, technique and treatment duration received by patients in this cohort is shown in Table 3. Median planned dose of radiation was 51.9 Gy over a median of 5.5 weeks, range 25.0 to 63 Gy. Comparing the planned versus delivered radiotherapy doses, we can see that the majority (98%) of patients received the planned dose of radiation. There was one recorded dose reduction, from 60 Gy to 12 Gy due to radiation complications (early moist desquamation).

**Table 3:** Radiation delivery method and dose received by patients in study (n = 66)

**Radiation technique**		**Number of patients treated n(%)**
3D-CRT		50 (76%)
IMRT		16 (24%)
**Radiation dose planned n(%)**		**Number of patients with dose reduction**
10-15 Gy	0 (0%)	0
15-20 Gy	0 (0%)	0
20-25 Gy	0 (0%)	0
25-30 Gy	3 (5%)	0
30-35 Gy	1 (2%)	0
35-40 Gy	0 (0%)	0
40-45 Gy	1 (2%)	0
45-50 Gy	6 (9%)	0
50-55 Gy	45 (70%)	0
55-60 Gy	9 (14%)	1 (Reduced to 12 Gy)
60-65 Gy	1 (2%)	0
**Median: 51.9 Gy**		
**Range: 25.0-63 Gy**		**Total: 1 (out of 66) (2%)**

The radiation dose received by patients in the study and dose reduction, both as a total number and as a percentage of study participants. Abbreviations: *3D-CRT* 3-Dimensional Conformal Radiation Therapy; *IMRT* Intensity Modulated Radiation Therapy; *Gy* gray.

Radiation techniques were also examined. Three-dimensional conformal RT (3D-CRT) was shown to be the primary technique used, with 50 patients (76%) receiving this treatment, while the remaining 16 patients (24%) received intensity-modulated RT (IMRT) (Table 3).

### Patient toxicities

Toxicity grades adverse events experienced by patients are described in Table 4 and include nausea, vomiting and stomatitis. A total of 54 patients (82%) experienced grades 0–1 toxicity, including minor changes in bowel habits, one episode of nausea and vomiting per day as well as the presence of painless ulcers, erythema or mild soreness. A total of seven patients (11%) experienced grade 2 toxicity, including moderate changes in bowel habits, 2–5 episodes of nausea and vomiting a day and painful erythema, edema or ulcers. Two patients (3%) experience grade diarrhea and three patients (5%) were classified as having grade 4 diarrhea.

**Table 4:** Adverse events experienced by patients in the study (n = 66)

**Toxicity grade of adverse event**	**Number of patients (percent) N = 66**
**0**	35 (53%)
**1**	19 (29%)
**2**	7 (11%)
**3**	2 (3%)
**4**	3 (5%)
**Case descriptions of grade 3 and 4 toxicity description (case by case)**	
Case 1, grade 3	Grade 2 stomatitis, grade 3 diarrhea
Case 2, grade 3	Grade 3 diarrhea, stool incontinence
Case 3, grade 4	Grade 4 diarrhea
Case 4, grade 4	Grade 4 diarrhea, stool incontinence
Case 5, grade 4	Grade 4 diarrhea

The adverse events experienced by patients in the study, including nausea, vomiting, stomatitis or diarrhea are shown in the top of the table. The lower half includes a more detailed description of each of the five grade 3 and 4 adverse events.

Peri-anal radiation dermatitis was experienced in varying degrees by numerous patients during the course of treatment (Table 5). Using the Dermatitis Grading Scale from the BCCA Care of Radiation Skin Reactions [21], there were a total of 19 patients (29%) that experienced grade 1 toxicities which encompassed minor skin changes such as numbness and tingling. Five patients (8%) experienced a grade two toxicity noted by erythema and swelling, and 42 patients (63%) experienced a grade 3 toxicity highlighted by instances of moist desquamation and ulceration.

**Table 5:** Radiation dermatitis experienced by patients in this study, by toxicity grade (n = 66)

**Toxicity grade**	**Number of patients (percent)**
**1**	19 (29%)
**2**	5 (8%)
**3**	42 (63%)

The breakdown of radiation dermatitis experienced by patients during this study based on toxicity grade.

### Patient outcomes

Median follow-up from time of initial diagnosis was 20 months. Of patients with Squamous cell histology (N = 62) 94% had no evidence of relapse as of April 2013. Four patients (6%), one stage I, one stage II and two stage III, had recorded relapses. One relapsed patient with initial stage II disease died, however, death was not attributed to treatment. Sorting the outcomes by cancer stage, one out of 26 patients (4%) with stage I tumours experienced a local relapse. For patients with stage II tumors, one out of 15 patients experienced a distant relapse, with two recorded deaths not attributed to treatment, and one out of the 25 patients with stage III tumours experiencing a distant relapse. Among 4 patients with tubulovillous adenoma and adenocarcinoma histology, 0% experienced a relapse.

## Discussion

In this study, the charts of 66 patients with stage I-III SCC of the anal canal treated at BCCA with multimodality therapy – Cap, MMC and radiation – were retrospectively reviewed. Although the global standard of care for SCC of the anal canal is concurrent radiotherapy with 5-FU and MMC, a standard protocol substituting infusional 5-FU with oral capecitabine was introduced in 2010 as an alternative non-infusional regimen [20]. Results of the study showed that while the majority of patients experienced some form of chemotherapy or radiation induced toxicity, protocol therapy was well tolerated; despite some dose reductions due to apparent toxicities there were no treatment discontinuations. The majority of patients who underwent this therapy since its initiation in 2010 currently have their tumors in remission with only a few reported cases of relapses and no treatment-related deaths.

Limitations encountered in this study centered on the retrospective nature of the collection of toxicity data. While the number of patients included was modest, the current study adds to the description of efficacy and outcomes of this regimen. Results compare favorably to the previous publication of the phase II study on which the current GICART protocol was based [19]. To our knowledge, there are no other prospective or retrospective studies describing this treatment regimen.

### Patient characteristics

Median age of patients in this cohort was 60 and the majority of tumors were stage I-II at presentation. It was noteworthy that there were almost twice as many female subjects in the study cohort as males. A significant increase in SCC has been documented in both men and women [22]. Most study subjects did not smoke, and there was only one HIV-positive subject included in the cohort. The preferred institutional chemotherapy regimen for HIV-positive patients is cisplatin-capecitabine due to the more favorable hematological toxicity described with cisplatin over MMC [6].

### Chemotherapy tolerance

Toxicity grades for nausea, vomiting and diarrhea were low despite the high dose intensity of chemotherapy drugs administered (Table 4). Most patients experienced only grade 0–1 toxicities indicating favorable outcomes with low side effects, while 12 patients (19%) experienced grade 2–4 toxicities for nausea, vomiting and diarrhea.

Overall, the starting doses of Cap (generally of 825 mg/m^2^ BID on each radiotherapy day) were well tolerated by the patients; however, 20% of patients required dose reductions, likely for reasons of apparent toxicity. A total of 8% of patients experienced grade 3–4 gastrointestinal toxicities which would lead to protocol-specified dose reductions. 11% experienced grade 2 gastrointestinal and skin toxicities which would require a dose reduction on second occurrence.

The chart review revealed that while all of the patients involved in this study experienced some degree of dermatitis during radiotherapy treatment, only one patient required radiation dose reduction. Generally, radiation treatment of any degree causes some sort of reaction in the treated and neighboring skin layers so the results themselves are not unexpected given the duration and intensity of the radiation doses.

### Radiation therapy

The GICART protocol specified 50.4 Gy in 28 fractions over 5.5 weeks. A range of treatment schedules, techniques and doses were used to reflect variability in individual patient factors and tumour characteristics. Generally, patients with a higher tumour stage or patients physically able to withstand higher doses of radiation were given larger radiation doses, while patients with lower stage tumors were treated with lower radiation doses. In a previous phase II study, escalating doses of radiation were prospectively defined in the following manner: patients with stage T1 tumours were given a dose of 45 Gy in 25 fractions, patients with stage T2 tumours were given a dose of 55 Gy in 30 fractions, and patients with stage T3–4 tumours were given 59 Gy in 32 fractions [23]. Radiation oncologists involved in the current study used similar criteria to justify dose variation according to tumor stage. There was no discernible difference in outcomes between the patients treated with the two radiation techniques, 3D-CRT and IMRT.

All patients included in the study achieved initial remission of disease. Subsequent relapses were infrequent and occurred in only 4% of stage I, 7% of stage II patients and in 6% of patients with stage III disease. Due to limited follow-up time, no conclusion regarding the efficacy of the regimen can be made at this time. However, phase III evidence from other disease settings show equivalence of capecitabine to infusional 5-FU in multiple settings [18,24,25]. One randomized head-to-head study of infusional 5-FU versus capecitabine in combination with radiation for stage II/III rectal cancer demonstrated equivalent efficacy and toxicity when given with pelvic radiation at doses of 50.4 Gy over 28–31 days [16,17].

No significant differences in the rates of pathologic complete response or grade 3–4 diarrhea were identified between the infusional 5-FU and versus CAP at a dose of 825 mg/m^2^ twice daily, 5 days/wk. Given the favorable tolerance and high dose intensity for both capecitabine and radiation described in the current study, it is unlikely that the substitution capecitabine for infusional 5-FU in the adjuvant treatment of SCC will result in inferior outcomes.

In this study there was a wide variation in radiation doses. At this point in time, the variations in administered radiation doses do not correlate with different outcomes, suggesting that lower radiation doses may be considered in some patients. Longer term follow up of patients in this cohort is necessary to ensure that patients who received lower doses of radiation and chemotherapy have similar outcomes to those who received larger doses. Our intention is to follow this cohort to see if additional correlations between radiation and chemotherapy and long term survival outcomes can be made.

## Conclusions

Combined modality therapy of capecitabine plus mitomycin and radiotherapy is well tolerated and allows high dose intensity of radiation and chemotherapy in a population based setting. Substitution of capecitabine for infusional 5-FU is feasible and may be a reasonable consideration for patients and physicians who prefer to avoid the inconvenience and potential complications of a central infusional device. A range of planned radiation doses, schedules and techniques were employed reflecting tumor and patient characteristics. Prospective studies to determine optimal radiation dose would be justified.

## Abbreviations

APR: Abdominoperineal resection; BCCA: British Columbia Cancer Agency; Cap: Capecitabine; HPV: Human papilloma virus; IMRT: Intensity-modulated RT; MMC: Mitomycin C; RT: Radiation therapy; SCC: Squamous cell carcinoma; 3D-CRT: Three-dimensional conformal RT; 5-FU: 5-fluorouracil.

## Competing interests

None of the authors have any conflicts of interest to report. Research support for this study was provided by the British Columbia Cancer Agency.

## Authors’ contributions

All authors, GT, BJ, MF and HFK, conceptualized the project, extracted and analysed data, and prepared the tables and manuscript. All authors read and approved the final manuscript.
